# Exposure to Patterned Auditory Stimuli during Acute Stress Prevents Despair-Like Behavior in Adult Mice That Were Previously Housed in an Enriched Environment in Combination with Auditory Stimuli

**DOI:** 10.1155/2018/8205245

**Published:** 2018-12-09

**Authors:** Enrique Flores-Gutiérrez, Edith Araceli Cabrera-Muñoz, Nelly Maritza Vega-Rivera, Leonardo Ortiz-López, Gerardo Bernabé Ramírez-Rodríguez

**Affiliations:** ^1^National Institute of Psychiatry “Ramón de la Fuente Muñiz”, Division of Clinical Investigations, Calzada Mexico-Xochimilco No. 101, C.P. 14370 Mexico City, Mexico; ^2^. National Institute of Psychiatry “Ramón de la Fuente Muñiz”, Laboratory of Neurogenesis, Division of Clinical Investigations, Calzada Mexico-Xochimilco No. 101, C.P. 14370 Mexico City, Mexico; ^3^National Institute of Psychiatry “Ramón de la Fuente Muñiz”, Laboratory of Neuropsychopharmacology, Division of Neuroscience, Calzada Mexico-Xochimilco No. 101, C.P. 14370 Mexico City, Mexico

## Abstract

Several interventions have been shown to counteract the effects of stress that may be related to improved neuroplasticity and neuronal activation. In this sense, environmental enrichment (ENR) protects against acute stress and increases neuroplasticity. It has been suggested that the use of patterned auditory stimuli (PAS) may be beneficial in increasing the effectiveness of ENR on disorders related to stress, such as depression and anxiety. Examples of PAS are classical music compositions that have interesting effects at both clinical and preclinical levels. Thus, we analyzed the effects of the exposure to PAS, represented in this study by Mozart's compositions, during ENR housing for 35 days in adult male Balb/C mice to evaluate depression-associated behavior using the forced-swim test (FST) paradigm with an additional short exposure to PAS. We found that the ENR mice that were exposed to PAS during both housing and behavioral task (ENR + PAS/FST + PAS) show decreased immobility and the number of despair episodes within a higher latency to show the first bout of immobility. Additionally, we found increased neuronal activation evaluated by the identification of activity-regulated cytoskeleton-associated protein- (Arc-) labeled cells in the prefrontal cortex (PFC) in mice exposed to PAS during housing and in the absence or presence of PAS during FST. Moreover, we found increased neuronal activation in the auditory cortex (AuCx) of mice exposed to PAS during FST. Our study suggests that the exposure to PAS during an emotional challenge decreases despair-like behavior in rodents that were previously housed in an enriched environment in combination with auditory stimuli. Thus, our data indicate that the role of the exposure to PAS as an intervention or in combination with positive environment to aid in treating neuropsychiatric disorders is worth pursuing.

## 1. Introduction

Environmental enrichment (ENR) has been widely described as strong social, physical, and cognitive stimuli that are able to induce beneficial effects in different types of preclinical models of neurodegenerative diseases, such as Parkinson's and Alzheimer's diseases (i.e., [1, 2]) and in preclinical models of psychiatric disorders, such as depression and anxiety (i.e., [3–5]). Thus, a wealth of data derived from animal studies reveals that ENR improves cognitive functions [6] and reverses anxiety- and depression-like behaviors that are induced by exposure to stress [3–5]. In fact, it has been shown that exposure to ENR after chronic stress can block the detrimental effects of stress on cognition [4, 5]. The mechanism by which ENR induces beneficial effects against depression- and anxiety-like behaviors in preclinical models is, in part, related to neuroplasticity-associated changes, such as the generation of new neurons in the dentate gyrus (i.e., [1, 2]). The presence of greater numbers of new neurons in the dentate gyrus can help to buffer the effects of stress (i.e., [3–8]). In addition, the protective effects of ENR have been related to increased levels of brain-derived neurotrophic factor (BDNF), nerve growth factor (NGF), and glia-derived neurotrophic factor (GDNF), which are implicated in neuroplasticity and neuronal activation processes [7–12]. Additionally, ENR increases the number of dendritic spines and the proportion of more mature dendritic spines in the dentate gyrus in rodents. Also, ENR favors learning and memory processes (i.e., [7–12]). In addition, some reports have suggested the involvement of signaling molecules, such as mitogen-activated protein kinases (MAPK) and protein kinase B, also known as Akt, in the beneficial effects of ENR (i.e., [12]).

Moreover, neuronal activation is indicative of the enrolment of neurons in information processing which is studied by examining the expression of immediate early genes including in them the activity-regulated cytoskeleton-associated protein (Arc) [13]. The immediate early genes Arc, cFos, or zif268 have relevant roles in processes including brain development, learning, and synaptic plasticity [14].

ENR in animal studies is composed of large cages with objects that vary in color and texture, tunnels and running wheels, and a large number of cagemates, which leads to high levels of sensory, cognitive, and social stimulation and physical activity [1, 2]. However, there is evidence indicating that ENR can be complemented with gustatory, olfactory, and auditory stimuli [2, 15, 16]. Regarding the latter, there is evidence from both rodent and human researches that indicates that patterned auditory stimuli (PAS) such as classical music induce a state of relaxation that leads to positive emotions [17, 18]. Thus, it has been suggested that the effects of music intervention are important to regulate behaviors including, but not limited to, emotion [19]. In fact, several studies have shown that music is able to induce favorable effects on numerous pathologies and in preclinical models, including, but not limited to, schizophrenia, anxiety, and depression [20–30]. For example, PAS exposure induced reduction in geriatric depression scores in humans [31], and it has been suggested that PAS may be useful to more effective and targeted treatments for depression [28].

Thus, a combination of PAS and ENR may be beneficial to increase the effectiveness of the treatments of such disorders [26–28]. Although both ENR and PAS are known to induce cognitive improvements, protect against stress or anxiety, and act as regulators of hippocampal neuroplasticity, including neurogenesis, the additive effect of PAS plus ENR on acute stress protection, structural neuroplasticity in the dentate gyrus of the hippocampus, or neuronal activation is not known. Thus, we hypothesized that the addition of PAS, such as compositions by Mozart, would increase the beneficial effects of ENR on neuroplasticity and neuronal activation and the protective effects of ENR on acute stress behavior in adult male Balb/C mice.

## 2. Materials and Methods

### 2.1. Animals

Seventy-three male Balb/C mice (mean age 8–10 months) were obtained from the animal facilities of the National Institute of Psychiatry “Ramón de la Fuente Muñiz”. Mice were housed in standard laboratory cages under a 12-hour light/12-hour dark cycle (starting at 0700 h and 1900 h, respectively) at a temperature of 23 ± 1°C with free access to food and water. All institutional and legal regulations regarding animal ethics, care, and handling were performed in accordance with the Mexican Official Norm for Animal Care (NOM-062-ZOO-1999) and approved by the local Institutional Ethics Committee of the National Institute of Psychiatry “Ramón de la Fuente Muñiz” (IACUC: CEI/02/06/20/09).

### 2.2. Behavioral Test Room

The animals were individually exposed to the forced-swimming test. All the behavioral procedures were conducted at the end of the dark phase of the light/dark cycle (ZT23) in a sound-isolated room. To minimize any possible influence of the time of exposure on behavioral tests, the experimental and control observations were alternated. The observer stayed in the same room, approximately 10 m away from the apparatus [32–34]. The tests were conducted under dim red light (44 lux).

### 2.3. Porsolt's Forced-Swim Test

Two days after the end of ENR and PAS housing, mice were exposed to Porsolt's forced-swim test (FST) [35], in the presence or absence of an additional PAS exposure. Mice were gently placed in a beaker (18 cm in diameter) that was filled to a depth of approximately 10 cm with water maintained at room temperature for a period of 6 min. After testing, each mouse was gently dried with a towel and placed in a heated cage with normal bedding covered by an absorbent paper towel for 30 min. Animals were then returned to their home cages for an additional 30 min prior to being sacrificed for neuronal activation analysis. The behavior of the animals was video recorded and analyzed blind to the experimental group with the ANY-maze behavioral tracking software (Stoelting Co., Wood Dale, IL, USA). The behavioral aspect detected was immobility detection (considering the minimal movements exerted by the animal to keep its head above water and floating). Thus, we analyzed the time spent immobile, number of episodes of immobility, and the latency to the first immobile episode. The period of time considered for immobility was 3000 milliseconds [35, 36].

### 2.4. Experimental Design

#### 2.4.1. Experimental Groups

**Figure 1:**
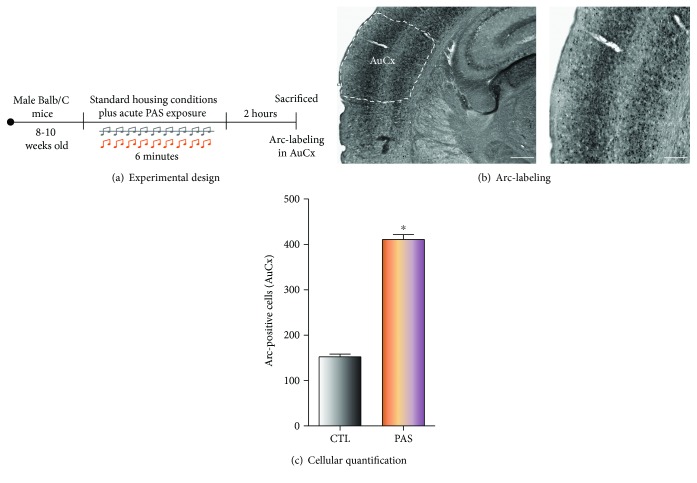
Experimental design and neuronal activation in the auditory cortex. (a) Mice were housed for 10 days under standard conditions. Next, rodents were exposed to PAS during six minutes and sacrificed two hours after PAS exposure to analyze Arc expression in the auditory cortex (AuCx). (b) Representative images of coronal sections within the AuCx are shown. High-power image exhibits the presence of Arc-labeled cells in the AuCx. Scale bars in (b) are equal to 150 and 50 *μ*m. (c) Cellular quantification shows an increase in number of Arc-positive cells in the AuCx after PAS exposure. Data represent the mean ± standard error of the mean (SEM). The unpaired Student *t*-test was used to compare both groups. The asterisk in (c) is equal to 0.049. *n* = 5 per group.

In the first experiment, animals were randomly assigned to one of the following groups: (a) normal housing without patterned auditory stimuli (control (CTL), *n* = 5) or (b) normal housing with patterned auditory stimuli (PAS, *n* = 5) applied for 6 minutes (Figure 1).

**Figure 2:**
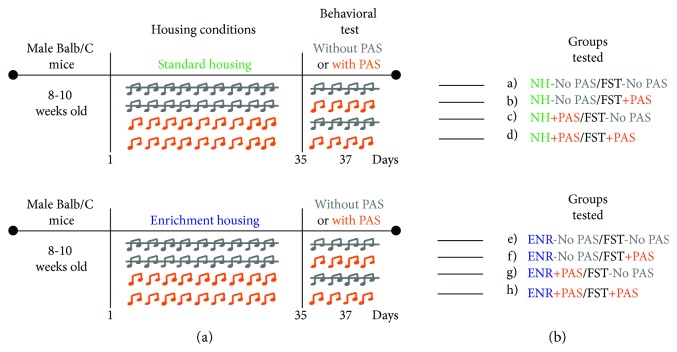
Experimental design to evaluate the effects of preexposure to standard conditions, environmental enrichment, patterned auditory stimuli, or environmental enrichment plus patterned auditory stimuli during housing and behavior in the forced-swim test. (a) Experimental design. Mice were randomly assigned to one of the following groups: (A) normal housing without patterned auditory stimuli and without patterned auditory stimuli during FST (NH-No PAS/FST-No PAS); (B) normal housing without patterned auditory stimuli plus PAS exposure during FST (NH-No PAS/FST + PAS); (C) normal housing with patterned auditory stimulus but without an additional PAS exposure during FST (NH + PAS/FST-No PAS); (D) NH + PAS plus an additional PAS exposure during FST (NH + PAS/FST + PAS); (E) ENR without patterned auditory stimuli and without patterned auditory stimuli during FST (ENR-No PAS/FST-No PAS); (F) ENR without patterned auditory stimuli plus PAS exposure during FST (ENR-No PAS/FST + PAS); (G) housed in ENR plus PAS but without an additional PAS exposure during FST (ENR + PAS/FST-No PAS); (H) ENR + PAS plus an additional PAS exposure during FST (ENR + PAS/FST + PAS). Two hours after the forced-swim test, the mice were sacrificed for histology. (b) Abbreviations of experimental groups.

In the second experiment, animals were randomly assigned to one of the following groups: (a) normal housing without patterned auditory stimuli and without patterned auditory stimuli during FST (NH-No PAS/FST-No PAS; *n* = 8); (b) normal housing without patterned auditory stimuli plus PAS exposure during FST (NH-No PAS/FST + PAS; *n* = 8); (c) normal housing with patterned auditory stimulus but without an additional PAS exposure during FST (NH + PAS/FST-No PAS; *n* = 8); (d) NH + PAS plus an additional PAS exposure during FST (NH + PAS/FST + PAS; *n* = 8); (e) ENR without patterned auditory stimuli and without patterned auditory stimuli during FST (ENR-No PAS/FST-No PAS; *n* = 8); (f) ENR without patterned auditory stimuli plus PAS exposure during FST (ENR-No PAS/FST + PAS; *n* = 8); (g) housed in ENR plus PAS but without an additional PAS exposure during FST (ENR + PAS/FST-No PAS; *n* = 8); and (h) ENR + PAS plus an additional PAS exposure during FST (ENR + PAS/FST + PAS; *n* = 7) (Figure 2).

#### 2.4.2. Environmental Enrichment

ENR was composed of large boxes (34 × 44 × 20 cm) containing tunnels of different colors and shapes, two running wheels, pieces of wood, nesting materials, and a small plastic house with stairs. To maintain novelty, the complexity of the tunnels was altered every third day. Control group mice were housed in normal cages. Housing conditions were maintained for 35 days.

#### 2.4.3. Patterned Auditory Stimuli

The PAS were applied in the dark phase of the natural light-dark cycle and generated from the signal amplification of a multimedia device, with a speaker system located 40 cm from the cage. The PAS were calibrated for an intensity of 70 ± 10 dB SPL, as previously reported (i.e., [37, 38]). Stimuli were given three times with a minimum of three hours between stimuli presentations. There were progressive increments in duration, from 9 min/d on the first day to 90 min/d at the end of the 5 weeks. The PAS application time was changed 5–15 minutes forward or backward each session to avoid time habituation. The stimulus consisted of pieces from Mozart, including chamber music, symphonic segments, concerts, and voice works. These pieces were played randomly, with different types each week, to avoid music habituation. All auditory stimuli were prepared with the software Audacity® 2.0.2 (open source, free, cross-platform for recording and editing sounds), at a sample rate of 44,100 per second. The Mozart pieces were transposed into the hearing range of mice using the Audacity® utility speed change, which altered time and frequency. We increased the tempo to 200% (e.g., an increase from 74 to 204 beats per minute) and shifted the frequency range from 7554 Hz to 19,485 Hz, which is within the auditory range of rodents [39] (Table 1).

### 2.5. Tissue Processing for Immunohistochemistry

Animals were sacrificed by an overdose of ketamine/xylazine (intraperitoneal, i.p.) followed by decapitation. Brains were removed and fixed with 4% *p*-formaldehyde (PFA) in 0.1 M phosphate buffer (pH = 7.4) for 7 days before they were stabilized in 30% sucrose in phosphate buffer. Serial coronal sections of 40 *μ*m were cut using a sliding microtome (Leica, Wetzlar, Germany) and stored at 4°C in a cryoprotective solution until required.

Brain coronal sections were incubated with primary antibodies for the immunodetection of doublecortin- (DCX-) positive cells or activity-regulated cytoskeleton-associated protein- (Arc-) positive cells in the dentate gyrus (DG), prefrontal cortex (PFC), and auditory cortex (AuCx). The antibodies used were goat anti-DCX (1 : 1000; Santa Cruz Biotech) and goat anti-Arc (1 : 5000; Synaptic-Systems), and the results were visualized using the peroxidase method [40, 41].

The number of DCX- or Arc-positive cells was determined in every 6th section from all animals [41, 42]. Cells positive for DCX or Arc were counted exhaustively using a 40x objective throughout the rostrocaudal extent of the granular cell layer (GCL; bregma −1.34 to −3.20). Counting was performed as previously described using the modified optical dissector method under bright-field light microscopy (Leica, Buffalo Grove, IL, USA). The cells appearing in the uppermost focal plane were excluded to avoid oversampling [42]. The resulting numbers were multiplied by 6 to obtain the estimated total number of DCX per granule cell layer.

In addition, Arc-positive cells were quantified in the PFC (bregma +2.10 mm to +1.78 mm) and in the AuCx (bregma −1.70 to −3.64) of six coronal sections per animal in areas of 0.264 ± 6.98 (PFC) or 221 ± 4.45 (AuCx) *μ*^2^. In the hippocampus, Arc-positive cells were quantified to report the number of Arc-positive cells per dentate gyrus (DG) (GCL; bregma −1.34 to −3.20). All samples were number coded, and the researcher conducting the analyses was blinded to the experimental groups.

### 2.6. ELISA for Plasma Corticosterone

Corticosterone levels were determined from plasma samples extracted by decapitation one hour after the FST. Blood (400 *μ*l) was collected in a small vial and kept on ice until centrifugation (950 *g* at 4°C) to allow for extraction of plasma. Tubes containing the plasma samples were protected with aluminium foil and kept at −80°C until analysis with a commercial ELISA kit (Enzo Life Sciences, Ann Arbor, MI USA). Levels of corticosterone were analyzed with a GloMax Discover ELISA reader (Promega).

### 2.7. Statistical Analysis

Analyses were performed using Prism 5.0 (GraphPad, San Diego, CA) or the SigmaStat 3.1 software (Systat Software Inc., San Diego, CA). For comparison between two groups, we applied unpaired Student *t*-test. Behavioral parameters, doublecortin, and neuronal activation were analyzed with a three-way ANOVA considering housing (factor A), presence or absence of PAS during housing (factor B) and presence or absence or presence of PAS during FST (factor C). This analysis was followed by two-way ANOVA and by the Student-Newman-Keuls post hoc test. Three-way ANOVA interactions that were not significant are shown in Table 2. The results are presented as the mean ± standard error of the mean (SEM). Differences were considered to be statistically significant at *p* ≤ 0.05.

## 3. Results

### 3.1. Short-Term Exposure to PAS Induces Neuronal Activation in the Auditory Cortex

First, to know whether adult male Balb/C mice were able to perceive the auditory stimuli used in the present study, animals were exposed to PAS to further quantify Arc-positive cells in the AuCx (Figure 1(a)). Quantification of Arc-positive cells in the AuCx (Figure 1(b)) revealed that mice exposed to PAS showed a significant increase (169%) in the number of Arc-positive cells compared to the control group (*p* = 0.049) (Figure 1(c)). This result suggests that adult male Balb/C mice respond to our PAS protocol.

### 3.2. Environmental Enrichment plus PAS Decreases Despair-Like Behavior in Mice Exposed to an Additional PAS during FST

**Figure 3:**
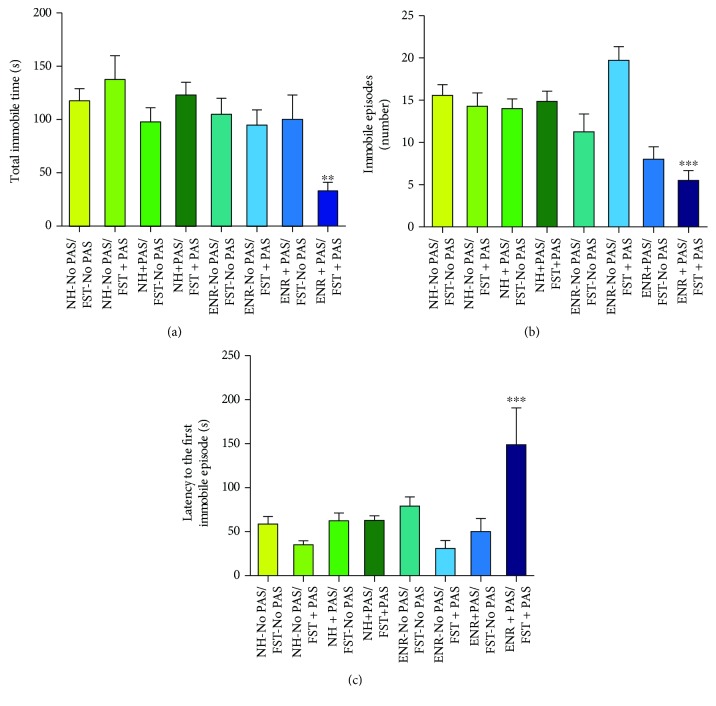
Effects of preexposure to standard conditions, environmental enrichment, patterned auditory stimuli, or environmental enrichment plus patterned auditory stimuli during housing and behavior in the forced-swim test. (a) Total time immobile, (b) immobile episodes, and (c) latency to the first immobile episode were analyzed. Data represent the mean ± standard error of the mean (SEM). Three-way ANOVA followed by the Student-Newman-Keuls test was performed, considering housing as factor A, presence of PAS during housing as factor B, and presence of PAS during FST as factor C. Asterisks in (a) indicate *p* = 0.01 (^∗∗^), in (b) indicate *p* < 0.001 (^∗∗∗^), and in (c) indicate *p* < 0.001 (^∗∗∗^) in ENR + PAS/FST + PAS compared with all other groups of mice. *n* = 7–8 per group.

In order to assess the protective effect of ENR plus PAS against acute stress, we quantified the time of immobility in the FST (Figures 2 and 3(a)). Post hoc comparison following a significant three-way interaction (housing (factor A), presence of PAS during housing (factor B), and presence of PAS during FST (factor C) (*F*_1,62_ = 4.88, *p* = 0.031)) revealed a significant decrease in the time of immobility in the ENR + PAS/FST + PAS mice compared with all other groups of mice (*p* = 0.01).

Additionally, we analyzed the number of immobile episodes (Figures 2 and 3(b)) during FST in male Balb/C mice. A significant three-way interaction (housing (factor A), presence of PAS during housing (factor B), and presence of PAS during FST (factor C) (*F*_1,62_ = 11.12, *p* = 0.002)) followed by post hoc comparison revealed a significant decrease in number of immobile episodes in the ENR + PAS/FST + PAS mice compared with all other groups of mice (*p* < 0.001).

Moreover, we analyzed the latency to the first immobile episode (Figures 2 and 3(c)) during FST in male Balb/C mice. Post hoc comparison following a significant three-way interaction (housing (factor A), presence of PAS during housing (factor B), and presence of PAS during FST (factor C) (*F*_1,62_ = 5.54, *p* = 0.022)) revealed a significant increase latency to the first immobile episode in the ENR + PAS/FST + PAS mice compared with all other groups of mice (*p* < 0.001). In addition, we found that corticosterone levels did not vary among the groups tested (data not shown).

These results strongly suggest that the exposure to PAS during behavioral challenge in the ENR mice that were housed for 35 days in the presence of auditory stimuli decreases despair-like behavior in male Balb/C mice.

### 3.3. Doublecortin-Labeled Cells in the Dentate Gyrus

**Figure 4:**
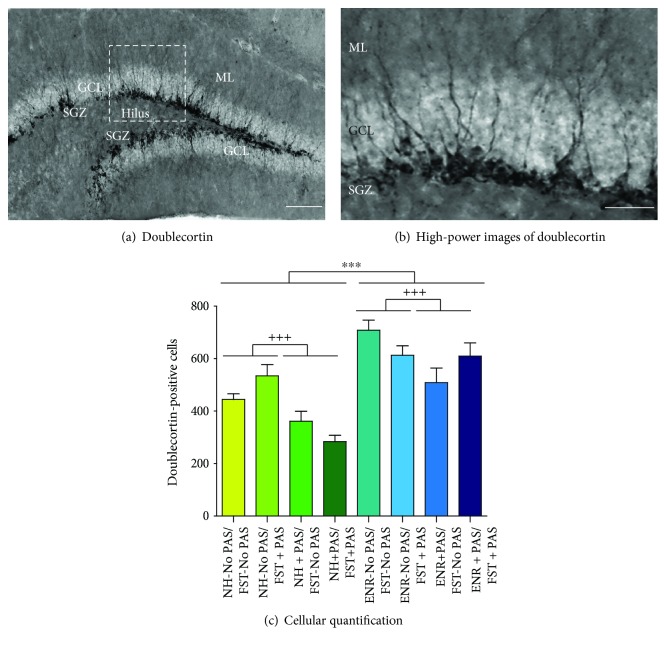
Effects of preexposure to patterned auditory stimuli on the number of doublecortin cells. (a) Representative images of coronal sections within the dentate gyrus showing DCX-labeled cells along the inner part of the granular cell layer (GCL). Images also show the subgranular zone (SGZ) of the dentate gyrus, the molecular layer (ML), and the hilus. (b) High-power image exhibits the presence of DCX-labeled cells in the dentate gyrus. Scale bars in (a, b) are equal to 150 and 50 *μ*m, respectively. (c) Number of DCX cells in each group. Data represent the mean ± standard error of the mean (SEM). Housing and PAS during housing exert significant main effects. Asterisks in (c) indicate *p* < 0.001 (^∗∗∗^) in ENR mice compared to normal housing mice. Crosses indicate *p* < 0.001 (^+++^) in groups of mice that were not exposed to PAS during housing compared to mice housed in the presence of PAS. *n* = 5 per group.

One of the important aspects that PAS and ENR modulate in terms of neuroplasticity is the generation of new neurons in the DG [1, 2]. New neurons have been related to buffer the effects of stress (i.e., [3–7, 9]). We here analyzed the number of DCX-associated cells which correspond to a population involved in the hippocampal neurogenic process (Figure 4) [40]. We found that significant main effects in the number of DCX-associated cells are caused by housing (*F*_1,39_ = 45.15, *p* < 0.001) and PAS during housing (*F*_1,39_ = 14.55, *p* < 0.001). Thus, ENR mice showed a higher number of DCX-associated cells compared to normal housing mice (*p* < 0.001). Moreover, the number of DCX-associated cells was higher in mice that were not exposed to PAS during housing than that found in mice housed in the presence of PAS (*p* < 0.001). The results indicate that the exposure to PAS during FST does not modify the number of DCX-associated cells induced with the ENR housing in the DG.

### 3.4. Neuronal Activation in the Dentate Gyrus following Despair-Like Behavior

**Figure 5:**
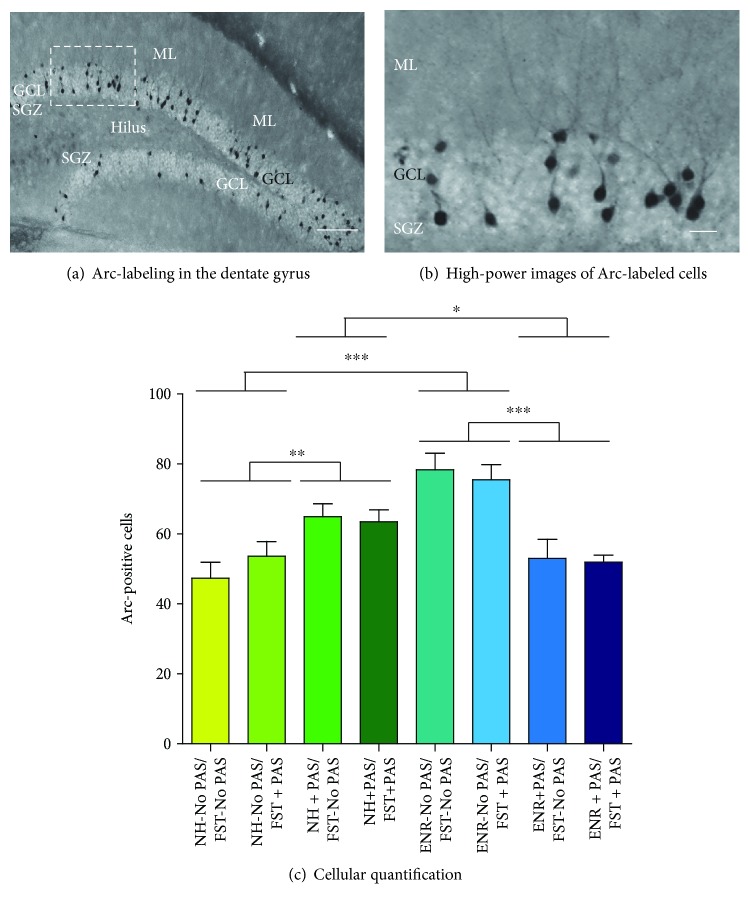
Effects of preexposure to standard conditions, environmental enrichment, patterned auditory stimuli, or environmental enrichment plus patterned auditory stimuli during housing and behavior on neuronal activation in the DG. (a) Representative images of coronal sections within the dentate gyrus showing Arc-labeling. Images also show the granular cell layer (GCL) of the dentate gyrus and the molecular layer (ML). (b) High-power image exhibits the presence of Arc-labeled cells in the GCL. Scale bars in (a, b) are equal to 150 and 30 *μ*m, respectively. (c) Number of Arc-labeled cells in the GCL. Data represent the mean ± standard error of the mean (SEM). Two-way ANOVA followed by the Student-Newman-Keuls test was performed, considering the housing as factor A and presence of PAS during housing as factor B. Asterisks in (c) indicate *p* = 0.007 (^∗^), *p* = 0.003 (^∗∗^), or *p* < 0.001 (^∗∗∗^). *n* = 5.

It has been proposed that the altered neuronal activation in the DG could be considered a “potential neurobiological biomarker for anxiety and depression and for their behavioral rescue” [43]. Considering this proposal, we assessed the neuronal activation of granule cells in the DG via Arc-protein labeling (Figure 5). Post hoc comparison following a significant two-way interaction of housing (factor A) and presence of PAS during housing (factor B) (*F*_1,39_ = 40.95, *p* < 0.001) indicated that the effect of PAS during housing within normal conditions caused an increase in the number of Arc-labeled cells in the DG (*p* = 0.003). However, the effect of PAS during housing within ENR showed a decrease in the number of Arc-labeled cells in the DG (*p* < 0.001). Also, ENR mice housed in the absence of PAS showed a higher number of Arc-labeled cells in the DG compared to the mice housed in normal conditions and in the absence of PAS (*p* < 0.001). However, mice housed in normal conditions and in the presence of PAS showed higher number of Arc-labeled cells in the DG compared to the ENR mice housed in the presence of PAS (*p* = 0.007).

### 3.5. Neuronal Activation in the Prefrontal Cortex

**Figure 6:**
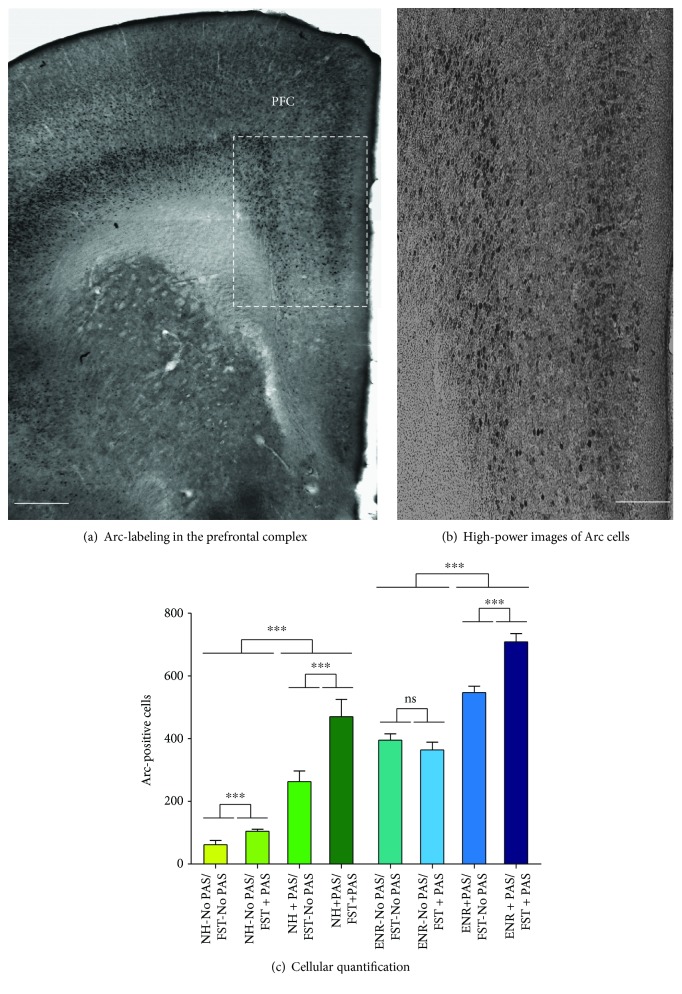
Effects of preexposure to standard conditions, environmental enrichment, patterned auditory stimuli, or environmental enrichment plus patterned auditory stimuli during housing and behavior on neuronal activation in the PFC. (a) Representative images of coronal sections within the prefrontal cortex (PFC) showing Arc-labeling. (b) High-power image exhibits the presence of Arc-labeled cells in the PFC. Scale bars in (a, b) are equal to 150 and 30 *μ*m, respectively. (c) Number of Arc-labeled cells in the PFC. Data represents the mean ± standard error of the mean (SEM). Two-way ANOVA followed by the Student-Newman-Keuls test was performed, considering the presence of PAS during housing as factor B and presence of PAS during FST as factor C. Asterisks in (c) indicate *p* < 0.001 (^∗∗∗^). *n* = 5.

We analyzed neuronal activation in the PFC, which is considered a key regulatory region and is implicated in a number of behaviors related to emotion (Figure 6) [44–46]. Post hoc comparison following a significant two-way interaction of the presence of PAS during housing (factor B) and presence of PAS during FST (factor C) (*F*_1,39_ = 23.13, *p* < 0.001) indicated that the effect of PAS during housing within the presence of PAS during FST caused an increase in the number of Arc-labeled cells in the PFC (*p* < 0.001). Also, the effect of PAS during FST in mice housed in the presence or absence of PAS showed an increase in the number of Arc-labeled cells in the PFC compared to the mice that were not exposed to PAS during FST (*p* < 0.001 and *p* < 0.001, respectively).

### 3.6. Neuronal Activation in the Auditory Cortex after Forced-Swim Test

**Figure 7:**
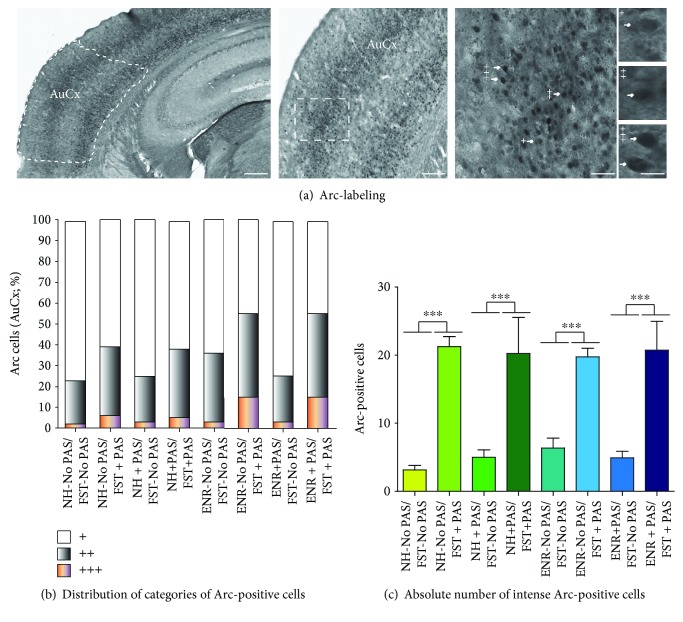
Effects of preexposure to standard conditions, environmental enrichment, patterned auditory stimuli, or environmental enrichment plus patterned auditory stimuli during housing and behavior on neuronal activation in the auditory cortex. (a) Representative images of coronal sections within the AuCx showing Arc-labeling. High-power image exhibits the presence of Arc-labeled cells in the AuCx. Scale bars in (a) are equal to 150, 75, 30, or 10 *μ*m. (b) Distribution of Arc-labeled cells in the AuCx. (c) Number of neurons Arc+++. Data represent the mean ± standard error of the mean (SEM, in (b–e)). Significant main effect is showed (*p* < 0001).

Finally, we corroborated the capability of mice to perceive PAS during the behavioral test via Arc-labeling in the AuCx (Figure 7). The distribution of Arc-positive cells in terms of intensity of labeling showed three categories (slight, intermediate, and intense) in all groups (Figures 6(a) and 6(b)). Interestingly, the ENR groups that were exposed to PAS during FST (ENR-No PAS/FST + PAS or ENR + PAS/FST + PAS) showed the highest percentage of Arc-positive cells corresponding to the intermediate (40%) and intense categories (15%) compared to the ENR-No PAS/FST-No PAS group or to the ENR + PAS/FST-No PAS group (intermediate: 33% and 22%, respectively; intense: 3% in both categories). Similar observations were found in the NH + PAS/FST + PAS mice (intermediate: 33% and intense: 5%, respectively) and in the NH-No PAS/FST + PAS group (intermediate: 33% and intense: 6%, respectively) compared to the NH-No PAS/FST-No PAS group or to the NH + PAS/FST-No PAS group (intermediate: 21 or 22% and intense: 2 or 3%, respectively).

Considering that there exist a higher proportion of activated neurons corresponding to the category of intense Arc-positive cells in mice exposed to PAS during FST, we calculated the number of intense Arc-positive cells in all of the groups (Figure 6(c)). Overall, we found that mice housed in NH or ENR conditions in the presence or absence of PAS during housing but exposed to PAS during FST showed the higher number of Arc-positive cells corresponding to the intense category in the AuCx (*p* < 0.001). Thus, the significant main effect in the number of Arc-positive cells is caused by the presence of PAS during FST (*F*_1,39_ = 100.49, *p* < 0.001). This result suggests that animals perceived PAS during the behavioral test.

## 4. Discussion

In the present study, we found that adult male Balb/C mice are able to perceive patterned auditory stimuli. Also, we confirmed that ENR produces structural neuroplasticity-related modifications in parallel with higher neuronal activation in the DG and PFC. Moreover, adult male mice preexposed to PAS do not show beneficial effects against acute stress reflected by despair-like behavior in the FST. However, we found that the preexposure to ENR plus PAS caused a higher neuronal activation in the PFC with a significant reduction in neuronal activation in the DG and despair-like behavior after FST. Similar effects were found in rodents that were preexposure to ENR plus PAS during housing but with an additional PAS exposure during the FST.

Environmental enrichment promotes neuroplastic- and neurochemistry-related changes with the potential to prevent or revert the effects of stress [1, 2, 4–6, 47]. Additionally, this paradigm favors learning and memory (i.e., [6, 41, 48]). Previous studies performed in different strains of mice have revealed that ENR also increases the levels of neurotrophins and growth factors [9, 10, 49]. In this study, we found similar results to those previously reported regarding how prehousing in an ENR can increase neuroplasticity (i.e., [47]), as reflected by the increase in DCX-associated neurons. It has been reported that the effects of ENR on neuroplasticity occur in parallel with the decrease in despair-like behavior in female mice (i.e., [47]).

Moreover, previous studies have proposed that the effectiveness of treatments for several disorders, including neuropsychiatric diseases, may be enhanced by other interventions, such as positive environmental activities, exposure to natural compounds, or exposure to patterned auditory stimuli [26, 27, 50, 51]. There are examples in the literature of the use of PAS, particularly (though not limited to) classical music, to induce cognitive improvement and protect against or reduce stress or anxiety symptoms, as well as for neuropsychiatric disorder interventions [20–30]. Among the PAS used in human and in rodent studies, Mozart's pieces have received attention for their effect on spatial memory (i.e., [52–54]). Although many studies exist demonstrating the benefits of exposure to PAS on behavior, the effects of PAS on structural neuroplasticity and the ability of PAS to enhance the benefits of positive environments, to our knowledge, are not known.

Here, we found that preexposure to PAS did not produce protection against the effects of a strong acute stress induced by a forced-swim test. This effect is contrary to the effects produced by other types of interventions, such as ENR or deep brain stimulation, the latter of which has also been shown to be effective against acute stress (i.e., [47, 55]). Moreover, in the present study, we found that PAS did not induce structural neuroplasticity-related changes in terms of the generation of new neurons associated to DCX in the DG of the hippocampus. However, neuronal activation after acute stress in mice preexposed to PAS or to an additional PAS during the FST increased in the DG, but in the PFC neuronal activation was importantly favored in rodents exposed to PAS during behavioral test although that increased number does not correlate with improvement behavior in the FST.

Interestingly, previous studies have reported the presence of neuronal hypoactivation in the DG of mice that exhibited despair-like behaviors under the emotional challenge induced by the forced-swim test [56]; this phenomenon was prevented by 21 days of treatment with the antidepressants fluoxetine or agomelatine [56, 57]. Considering this effect, Sah and collaborators proposed that the altered neuronal activation in the DG could be considered a “potential neurobiological biomarker for anxiety and depression and for their behavioral rescue” [56]. This proposal is in agreement with a previous study that showed hippocampal hypoactivation in patients with major depressive disorder under challenge conditions [43]. In agreement with this asseveration, the ENR group housed in the absence of PAS exhibited a higher activation in the DG and in the PFC. Surprisingly, the addition of PAS to the ENR housing did not significantly increase the beneficial effects of ENR on structure-related neuroplasticity or on despair-like behavior compared with the mice that were subjected to standard housing plus PAS. However, preexposure to PAS in the ENR mice reduced neuronal activation in the DG, an effect that was not observed under standard-housing conditions plus PAS. Thus, these observations may suggest that PAS interfere with an increase in neuronal activation that was caused by ENR in the DG. Similar effects were observed after exposure to PAS but during the FST in the ENR mice. Also, a significant decrease was observed for parameters that were related to despair-like behavior. In addition, we found a slight decrease in neuronal activation in the DG and a greater increase in neuronal activation in the PFC. Interestingly, the exposure to PAS without ENR during housing also induced an increase in neuronal activation in the PFC; this effect was more evident when the mice were exposed to a second period of PAS during the behavioral task. However, this exposure did not impact the parameters that were related to despair-like behavior, which suggested that ENR induced modifications on neuroplasticity at different levels or involved different neuronal ensembles that may favor the effect of PAS in increasing ENR benefits during exposure to the acute-stress paradigm.

The PFC is a key regulatory region implicated in a number of behaviors, including working memory, decision-making, goal-directed behaviors, and social behaviors [44–46]. Additionally, the PFC is connected with several cortical and subcortical regions of the brain, including the nucleus accumbens, amygdala, and hypothalamus and regions of the cortex that process motor and sensory inputs and motor responses [58–60]. In this sense, it has been recently reviewed that the AuCx connects and underlies some functions of the PFC [61]. Here we showed that PAS induced increase neuronal activation reflected by Arc-labeled cells of the three different categories that revealed that independently of the prehousing conditions, mice were able to perceive the auditory stimuli during the behavioral challenge with the strongest effect on the category of intense Arc-labeled cells. Interestingly, it has been shown that the PFC in humans is activated after hearing classical music [62]. Moreover, PFC is influenced by patterned auditory stimuli because it is connected to inner structures of the brain such as structures of the limbic system such as the amygdala and hippocampus, brain regions implicated with emotional regulation [63].

Thus, the data derived from our study provide support to previous reports that suggest that the limbic areas show connectivity and are activated or deactivated to counteract the effects of acute stress [64–68]. In this regard, the stronger connectivity between the PFC and the DG is important to improve adverse behavioral inhibition [69–71]. Also, our results regarding the effects of PAS and ENR may indicate the importance of the PFC in adapting to challenging situations (i.e., [69–71]). Although this assessment is interesting, it should be evaluated in a specific study with a more complete battery of behavioral tests. However, it is important to note that ENR induces neuroplastic- and neurochemistry-related changes to cope against the effects of stress [1, 2, 4–6, 47]. Thus, ENR could make the brain more flexible (i.e., [48]) to favor its capability to perceive patterned auditory stimuli under challenge conditions to deal successfully with stress. Moreover, it is important to note that the effects observed in our study cannot be explained by significant changes in the hypothalamic-pituitary-amygdala (HPA) axis in terms of the levels of corticosterone; thus, additional studies may be necessary to study additional markers of HPA axis. Also, our study cannot explain the beneficial effects of PAS in ENR mice exposed to an additional PAS during FST by increased levels of BDNF nor by increased levels of DCX neurons, as was previously reported in Sprague–Dawley rats exposed to PAS from the postnatal day 1 [54].

## 5. Conclusion

Finally, our study shows that preexposure to PAS does not exert a reversal of despair-like behavior in parallel with the differential neuronal activation of the PFC without affecting activation in the DG. Moreover, the combination PAS-ENR with a short exposure to an additional PAS episode during an emotional challenge, represented in this study by the FST, exhibited a higher protection against adverse behavioral challenges, such as acute stress with increased neuronal activation in the PFC and with decreased neuronal activation in the DG. Thus, it is possible that ENR promotes flexibility of the brain to learn (i.e., [48]) patterned auditory stimuli, which may be perceived and evoked under challenge conditions, to deal successfully with stress. Although this idea is suggestive, it remains to be tested. Nevertheless, our data indicate that ENR housing together with PAS prevents the occurrence of despair-like behavior when an additional exposure to PAS is present during the behavioral task in male adult Balb/C mice. However, it is worth pursuing the medical relevance of PAS as an intervention or in combination with positive environmental modifications to aid in treating depression.

## Figures and Tables

**Table 1:** Presentation of the patterned auditory stimuli during housing of young male Balb/C mice.

Week^∗^	Increase stimulation per week (minutes; min)^∗∗^	Number of pieces applied in random order^∗∗∗^
First	01 to 07	8
Second	08 to 15	9
Third	16 to 21	10
Fourth	22 to 30	13
Fifth	30	48
Sixth	30	88

^∗^The week form Thursday including Saturdays and Sundays. ^∗∗^There were three sessions each day during the dark phase. Duration of stimuli changes about one or two minutes from one session to another. ^∗∗∗^We applied different works for each week. This difference corresponded to at least 80% of the works. All PAS works were accelerated 200% in tempo and frequency as is indicated in Materials and Methods.

**Table 2:** Three-way ANOVA of parameters that do not showed significant interactions.

Parameter	Factors	Results	Interaction	Results
Doublecortin-labeled cells	Housing (A)	*F*_1,39_ = 45.15, *p* < 0.001	AxBxC	*F*_1,39_ = 6.13, ns
	PAS during housing (B)	*F*_1,39_ = 14.55, *p* < 0.001		
	PAS during FST (C)			
		*F*_1,39_ = 0.04, *p* = 0.82		
Neuronal activation in the dentate gyrus	Housing (A)	*F*_1,39_ = 6.03, *p* = 0.024	AxBxC	*F*_1,39_ = 0.65, ns
	PAS during housing (B)	*F*_1,39_ = 3.25, *p* = 0.087		
	PAS during FST (C)	*F*_1,39_ = 0.005, *p* = 0.94		
Neuronal activation in the prefrontal cortex	Housing (A)	*F*_1,39_ = 226.28, *p* < 0.001	AxBxC	*F*_1,39_ = 0.15, ns
	PAS during housing (B)	*F*_1,39_ = 205.82, *p* < 0.001		
	PAS during FST (C)	*F*_1,39_ = 26.34, *p* < 0.001		
Neuronal activation in the auditory cortex	Housing (A)	*F*_1,39_ = 0.29, *p* = 0.59	AxBxC	*F*_1,39_ = 0.54, ns
	PAS during housing (B)	*F*_1,39_ = 0.0079, *p* = 0.92		
	PAS during FST (C)	*F*_1,39_ = 100.49, *p* < 0.001		

## Data Availability

The data used to support the finding of this study are included within the article.
